# ‘From women for women’: A citizen science approach engaging women in the isolation and application of the vaginal health-associated bacterium *Lactobacillus crispatus*

**DOI:** 10.1371/journal.pone.0308526

**Published:** 2024-11-11

**Authors:** Shardelice Illidge, Remco Kort, Rosanne Hertzberger, The Dutch crispatus Citizen Science Collective

**Affiliations:** 1 Amsterdam Institute for Life and Environment (A-LIFE), Vrije Universiteit Amsterdam, Amsterdam, The Netherlands; 2 Stichting *crispatus*, Amsterdam, The Netherlands; 3 ARTIS-Micropia, Amsterdam, The Netherlands; Universidad San Francisco de Quito, ECUADOR

## Abstract

A vaginal microbiome rich in *Lactobacillus crispatus* is associated with good reproductive and sexual health outcomes. Dysbiosis, indicated by the loss of *Lactobacillus crispatus*, is a risk factor for urogenital infections, such as the clinical diagnosis of bacterial vaginosis (BV) or urinary tract infections. While many scientists have explored probiotics using a conventional pharmaceutical approach, concerns about accessibility and affordability prompt an investigation into a preventive approach using this naturally occurring bacterium. Our study aimed to explore a potential woman-friendly vaginal probiotic product using the naturally occurring bacterium, *Lactobacillus crispatus*. Citizen scientists actively participated in a two-day practicum and successfully performed the procedures using self-collected vaginal swabs. The practicum received positive responses from participants who demonstrated notable engagement and enthusiasm. With expert guidance, participants without a laboratory background were able to execute assigned tasks successfully. From the Dutch *crispatus* Citizen Science Collective of 48 women, 22 succeeded in isolating their own *Lactobacillus crispatus* strains using a Loop-Mediated Isothermal Amplification (LAMP) protocol for identification. Additionally, 48 metagenomes and 54 whole genomes from 22 individuals were sequenced for comparative analysis. This project effectively engaged a community of women in the isolation of *Lactobacillus crispatus* strains from their vaginal microbiota, followed by *in vitro* characterization experiments and a hackathon for the development of a probiotic product. Our citizen science approach opens up collaboration possibilities and new avenues for exploration of vaginal health, facilitating community involvement and the development of targeted interventions to enhance women’s well-being.

## Introduction

Studies have shown that a vaginal microbiome rich in *Lactobacillus crispatus* is associated with good reproductive and sexual health outcomes, such as a reduced risk of preterm births and urogenital infections. These bacteria are able to degrade and grow on glycogen present in the vagina [1, 2], and produce inhibitory substances such as lactic acid, hydrogen peroxide and bacteriocins [3], which curbs the growth of other organisms that would otherwise cause an infection [4].

A loss of *Lactobacillus* dominance indicates a state of dysbiosis, that is, an overgrowth of anaerobic bacteria and high species diversity in the vagina. Dysbiosis is a risk factor for urogenital infections such as the clinical diagnosis of bacterial vaginosis (BV) or urinary tract infection [5–7]. BV is associated with an increased risk of acquiring and transmitting not only bacterial STD’s such as Chlamydia and Gonorrhea, but also viral infections, such as HIV, HSV, and HPV [7–9]. While a diverse collection of bacteria is desirable for good health, in the gut, the inverse is true for the vagina [7, 10].

Antibiotics are often prescribed to women experiencing adverse health effects owing to imbalances in the vaginal microbiome. Many scientists have explored the potential of probiotics, which are live biotherapeutic products based on bacteria, such as *L*. *crispatus*, as an alternative treatment for BV. This shift is motivated by concerns regarding the disruptive effect of antibiotics on the healthy microbiome of the body [11]. These probiotics are currently being developed using a conventional pharmaceutical approach. As a result, these products are protected by intellectual property, financially supported by venture capital, and geared towards distribution through prescription-dependent medical channels.

The pharmaceutical route raises concerns regarding accessibility and affordability for women seeking the effective prevention of serious health problems. We also question the appropriateness of this approach for a product that utilizes a naturally occurring bacterium abundantly present in the bodies of women and is likely to have a preventive rather than a curative effect.

The primary goal of this project is to gain a deeper understanding of the bacteria in the vaginal flora, specifically focusing on the subtle differences among *L*. *crispatus* bacteria. Additionally, we aim to collect *L*. *crispatus* bacteria to investigate the possibility that these bacteria colonize the vagina of other women who do not possess the bacteria. Based on previous studies, we anticipate that these bacteria may help prevent infections [12]. We aim to create a woman-friendly vaginal probiotic that could help prevent urogenital infections by developing a multi-strain over-the-counter probiotic that is accessible to everyone and has long-lasting colonization.

For this purpose, we asked citizen scientists to culture, identify, and isolate *L*. *crispatus*. We conducted a two-day practicum during which participants collected vaginal swabs, examined their own vaginal microbiota under a microscope, inoculated agar plates, and after three to seven days of incubation, received guidance in recognizing *L*. *crispatus* colonies. Subsequently, utilizing the loop-mediated isothermal amplification method (LAMP) [13, 14], they identified and isolated colonies of vaginal *L*. *crispatus* strains on fresh agar plates.

To account for their research, citizen scientists collectively constitute a group of authors. Additionally, participants form a donor panel within the *crispatus* foundation that has the right to, among other things, accept or reject new sublicences of isolated *L*. *crispatus* strains per majority vote. They were also invited to participate in a hackathon aimed at developing a probiotic product. Together, these aspects establish a platform where the lived experiences of donors inform the collaboration between them and scientists, which in turn fosters response-ability in relation to *L*. *crispatus* bacteria and future users of vaginal probiotics.

## Materials and method

Human participants for the citizen science in this study were recruited from April 30 to September 1, 2023. A written informed consent was obtained from all participants. Citizen scientists actively participated in a two-day practicum, successfully performing procedures with self-collected vaginal swabs, including the isolation of *L*. *crispatus* by selective enrichment on nutrient agar plates, species identification by Loop-Mediated Isothermal Amplification (LAMP), microscopy, and pH measurements. The detailed protocol described in this peer-reviewed article is published on protocols.io, [dx.doi.org/10.17504/protocols.io.81wgbzdkygpk/v1] and is includedas supporting information file 1 with this article.

### Ethics statement

The Medical Ethics Committee (METC) of the Vrije Universiteit, Amsterdam reviewed the research protocol (Reference 2022.0791) and concluded that the Dutch Medical Research Involving Human Subjects Act—in Dutch: wet medisch-wetenschappelijk onderzoek met mensen (WMO) does not apply to this research protocol (‘not subject to WMO’).

## Conclusion

The citizen scientists successfully cultivated, identified, and isolated *L*. *crispatus* following the instructions provided to them. We isolated *L*. *crispatus* strains from 22 of 48 women aged between 18 and 45 years. This achievement underscores that with proper guidance, we can make certain scientific practices accessible to a wider audience without compromising the quality of research.

This citizen science project raised awareness of vaginal health within the community and fostered open discussions between scientists and participants. Moreover, it successfully encouraged enthusiastic engagement from participants, whose feedback concretely shaped the practicum, license agreement, and this article. As a result, our citizen science approach opened up collaboration possibilities and new avenues for exploration of vaginal health, facilitating community involvement and the development of targeted interventions to enhance women’s well-being.

## Expected results

Research findings have highlighted a correlation between the abundance of *L*. *crispatus* and female reproductive hormone levels. Given that estrogen and progesterone levels significantly decrease in postpartum women, we hypothesized that isolating *Lactobacillus crispatus* during this period would be challenging [15]. This pattern was also observed in postmenopausal women.

A study conducted in Amsterdam on the prevalence of *Lactobacillus* dominance in the vaginal microbiota of women aged 18–34, indicated that approximately 60% of the population was dominated by *Lactobacillus* spp, including *L*. *crispatus*, *L*. *ine*rs, *L*. *gasseri*, and *L*. *jensenii* [16]. We expected similar results in the present study.

We also understand that low vaginal pH, attributed to the production of lactic acid, along with the observation of rod-shaped bacteria under light microscopy, serves as an indicator of the presence of *Lactobacillus* spp.

This citizen-science-based lab protocol allows the isolation of *Lactobacillus crispatus* strains from women, followed by the characterization of inter- and intra-individual differences between genotypes and phenotypes of these strains and product development for vaginal health. This citizen science initiative including a cultivation-based approach for the isolation of *Lactobacillus crispatus* is complementary to a recent initiative that included cultivation-independent analysis of the vaginal microbiomes of 3,345 women, leading to a the further understanding of the composition and function of the vaginal microbiome [17].

## Supporting information

S1 File Step-by-step protocol, also available on [dx.doi.org/10.17504/protocols.io.81wgbzdkygpk/v1]. (PDF)
