# Supplementary material for: ‘From women for women’: A citizen science approach engaging women in the isolation and application of the vaginal health-associated bacterium Lactobacillus crispatus
Source: PLoS One. 2024 Nov 11;19(11):e0308526. doi: 10.1371/journal.pone.0308526 (PMC11554043; doi:10.1371/journal.pone.0308526)
Supplement: S1 File — (PDF) [file pone.0308526.s001.pdf]

Apr 15, 2024

‘From women for women’: A citizen science approach engaging women in the isolation and application of the vaginal health-associated bacterium Lactobacillus crispatus

DOI
dx.doi.org/10.17504/protocols.io.81wgbzdkygpk/v1

Shardelice Illidge[1], Remco Kort[1,2,3], Rosanne Hertzberger[1,2]

[1]Amsterdam Institute for Life and Environment (A-LIFE), Vrije Universiteit Amsterdam, The Netherlands; [2]Stichting crispatus, Amsterdam, The Netherlands; [3]ARTIS-Micropia, Amsterdam, The Netherlands

Remco Kort
Amsterdam Institute for Life and Environment (A-LIFE), Vrije...

OPEN ACCESS

DOI: dx.doi.org/10.17504/protocols.io.81wgbzdkygpk/v1

Protocol Citation: Shardelice Illidge, Remco Kort, Rosanne Hertzberger 2024. ‘From women for women’: A citizen science approach engaging women in the isolation and application of the vaginal health-associated bacterium Lactobacillus crispatus. **protocols.io** https://dx.doi.org/10.17504/protocols.io.81wgbzdkygpk/v1

License: This is an open access protocol distributed under the terms of the **Creative Commons Attribution License**, which permits unrestricted use, distribution, and reproduction in any medium, provided the original author and source are credited

Protocol status: Working
We use this protocol and it's working

Created: March 05, 2024

Last Modified: April 15, 2024

Protocol Integer ID: 97133

Keywords: Vaginal health, probiotic, Lactobacillus crispatus, citizen science

Funders Acknowledgement:
TKI-project Citizen Science for Health Care
Grant ID: 404270598028

Abstract

A vaginal microbiome rich in *Lactobacillus crispatus* is associated with good reproductive and sexual health outcomes. Dysbiosis, indicated by a loss of *Lactobacillus crispatus*, is a risk factor for urogenital infections such as the clinical diagnosis of bacterial vaginosis (BV) or urinary tract infections. While many scientists explore probiotics using a conventional pharmaceutical approach, concerns about the accessibility and affordability prompt an investigation into a preventive approach using this naturally occurring bacterium. Our study is aimed at the exploration of a potential woman-friendly vaginal probiotic product by use of the naturally occurring bacterium *Lactobacillus crispatus*. Citizen scientists actively participated in a two-day practicum, successfully performing procedures with self-collected vaginal swabs. The practicum received a positive response from participants, who demonstrated notable engagement and enthusiasm. With expert guidance, participants without a laboratory background were able to successfully execute the assigned tasks. From the Dutch *crispatus* Citizen Science Collective of 48 women, 22 succeeded to isolate their own *Lactobacillus crispatus* strains, using a Loop-Mediated Isothermal Amplification (LAMP) protocol for identification. In addition, 48 metagenomes and 54 whole genomes from 22 individuals were sequenced for comparative analysis by an external company. This project effectively engaged a community of women into isolation of *Lactobacillus crispatus* strains from their vaginal microbiota followed by in vitro characterization experiments and a hackathon for the development of a probiotic product. Our citizen science approach opens up collaboration possibilities and new avenues for exploration in vaginal health, facilitating community involvement and the development of targeted intervention to enhance women's well-being.

Attachments

PDF

pyxxcb5bx.pdf

1.7MB

Guidelines

Setting up a women's collective:

- The ideas for the citizen science initiative described here were presented to "The *crispatus* foundation" to manifest its vision and curate a collection of *L. crispatus* isolates. Participants contribute their isolates under a licensing agreement, allowing the foundation to negotiate sub-licensing for probiotic product development. (Supplemental file 1: Appendix A. Licensing agreement).
- Participants retain ownership of their contributions, ensuring their intellectual property rights are safeguarded. Donors, upon joining a panel, gain oversight and voting rights concerning sub-licensing agreements. They are entitled to receive 20% of the profits generated, while the remaining 80% of the foundation's profits are dedicated to funding new research initiatives.
- For privacy concerns, every participant chose their own pseudonym. Zivver (Zivver, Amsterdam, The Netherlands), a platform that allows the exchange of information in an easy and secure way, was used for sharing sensitive information during digital communication.

Recruitment:

To keep the sample size closer to our target number, recruitment was mostly based on word of mouth, newsletters, a small publication in a local newspaper or a flyer on a college campus. Initially, we attracted a diverse group of women interested in participating in the study, all with different backgrounds and life experiences spanning ages 18 to 75. As we progressed, however, challenges in collecting *L. crispatus* from women aged 40 and above led us to refine the criteria, focusing on women between the ages of 18 and 40. We had a total of five practicals with 48 participants. We were able to isolate *L. crispatus* from 22 women. The practicals were held at three different locations in The Netherlands: Vrije Universiteit Amsterdam, Avans Hogeschool, Breda and BioARTLaboratories Eindhoven.

Before start

This protocol can be followed in a low-resource environment. All volunteers participating in the initiative must sign a consent form, ensuring they are both willing and legally competent to understand the implications of their involvement. It is paramount that privacy is upheld and guaranteed at all times, safeguarding the confidentiality of those involved.

Practicals

6h

1 We organised a two-day practical with two hour sessions each day. Upon registration, we shared additional information with potential donors about the importance and the reason for our project (Supplemental file 2: Appendix B. Participant information).

2 Participants received an online questionnaire (Supplemental file 3: Appendix C. Participation in the *crispatus* Study - Questionnaire, that focused on hormonal aspects, and a sampling kit at their home address containing:

2.1 Two sample collection tubes. One for metagenomic sampling (with DNA/RNA shield buffer), and one for microscopy and isolation (e-swab with Amies transport medium).

2.2 A swab for a pH self-test.

2.3 An information booklet that also served as a personal lab journal.

3 The contents of the sampling kit that a donor would receive are illustrated in Figure 1.

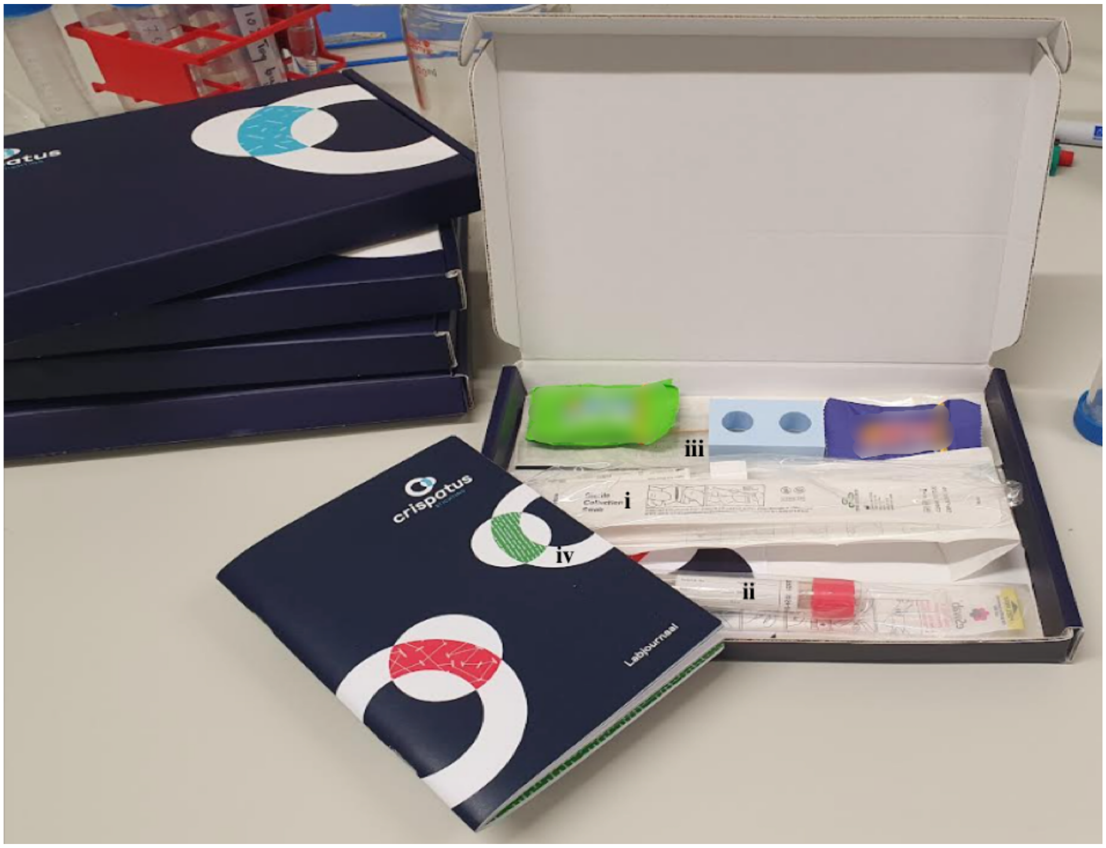

Figure 1: Content of a sampling kit, containing i) one collection tube with DNA/RNA shield buffer for metagenomic sampling; ii) one collection tube (e-swab) with Amies transport medium for isolation and microscopy; iii) a swab for a pH self-test; and iv) an information booklet that also served as a personal lab journal. Picture created by Rosanne Hertzberger, 2023.

4 The women were advised to take the swabs a maximum of four hours before the start of the practical (Supplemental file 4: Appendix D. Section on Swab Sampling from the Lab Journal).

- Taking the swabs on location is also an option. We used a lactation room or bathrooms for this purpose.
- A short introduction is given about the purpose of the project and how we go about achieving our objective.
- Since the practical is taking place in a laboratory, basic safety lab instructions are given to the women.

5 The informed consent and licensing agreement were discussed and signed before starting the practical (Supplemental file 5: Appendix E. Consent form *crispatus* practical). The women were also informed that they could stop research participation at any time without having to give any reason for it.

Microscopy

6 Citizen scientists fixed and stained a droplet from the e-swab collection tube on a microscope slide and were assisted to visualise their vaginal sample at 1000x magnification. Epithelial cells

and lactobacilli were easily identified. Participants made descriptions of their samples in their lab journal and received a printed screenshot, as illustrated in Figure 2. See Supplemental file 6: Appendix F. Section on Microscopy from the Lab Journal, for the instructions provided to the donors.

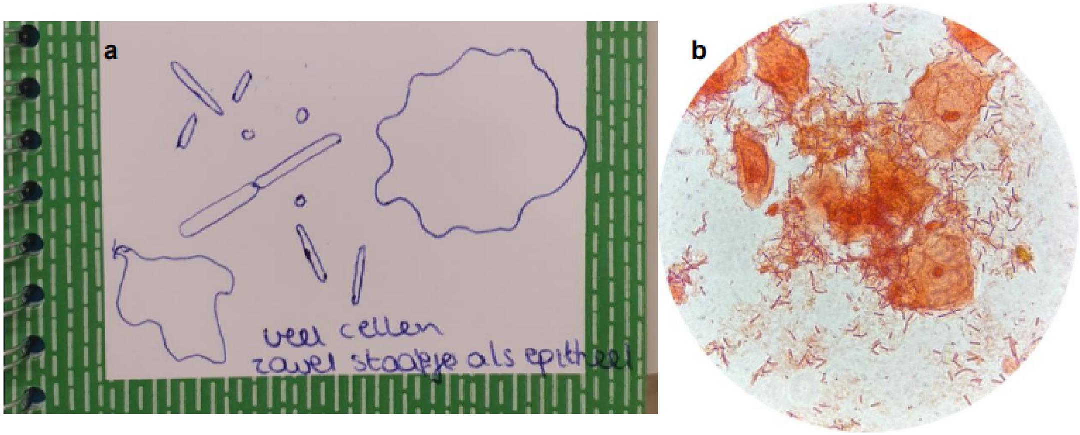

Figure 2: a) Illustrated description of microscopic view in a lab journal. b) Microscopic picture of vaginal sample showing large vaginal epithelial cells with a cell nucleus and small rod-shaped Lactobacillus bacteria.

Lactobacillus crispatus colony identification using a colorimetric test

30m

7 For the identification of *L. crispatus* colonies we need to implement a microbial molecular technique that will be easy for the general public to carry out without the need for a microbiology training.

7.1 We used a *L. crispatus*-specific Loop-mediated isothermal amplification protocol (LAMP) as an alternative to colony PCR to allow participants to identify strains of *L. crispatus* [1].

7.2 Supplemental file 7: Appendix G. Section on Identification and Isolation from the Lab Journal, provide instructions to the donors and Supplemental file 8: Appendix H. Loop-mediated isothermal amplification protocol, for the protocol.

8 The LAMP is a DNA amplification method with high specificity and amplification efficiency. It allows the reaction to take place at a constant temperature, 65 °C , within 00:30:00 . 30m

8.1 It uses 4 primers and a DNA polymerase with strand displacement activity. The WarmStart® Colorimetric Master Mix (New England Biolabs, Ipswich, Massachusetts, United States) that is used in this protocol, has a low Tris buffer concentration at pH 8 .

8.2 The mix contains a pH indicator, phenol red, that changes colour from red to yellow below a pH of pH 6.8 , as shown in Figure 3 [2].

- This colour change occurs due to a drop in pH by protons that are released during the amplification process [3].

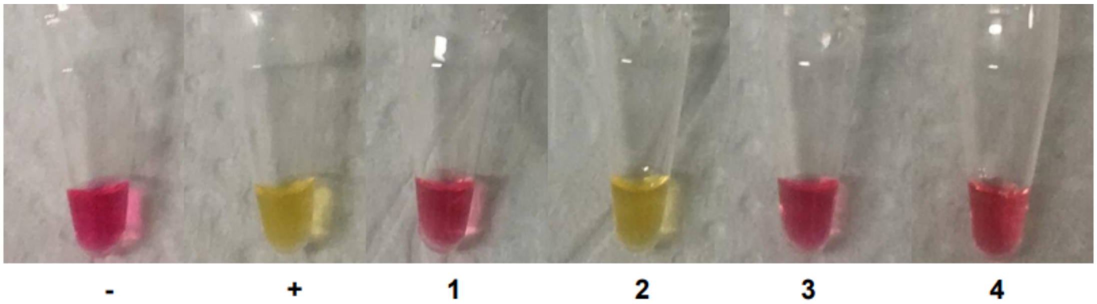

Figure 3: Validation results of the Loop-mediated isothermal amplification method test for the citizen science practical. - negative controle, + positive controle, 1 *L. plantarum*, 2 *L. crispatus*, 3 *L. delbrueckii* sub sp.*bulgaricus*, 4 *L. gasseri*.

9 A previous study designed unique LAMP primers for *L. crispatus* and it showed that the LAMP test has a detection limit of 10 fg DNA [1].

- This was followed up by a dilution step in the protocol with 100 µL Milli-Q to dilute any lactic acid associated with the colony and prevent false positives.

10 The LAMP test was validated using colonies of *Lactobacillus plantarum*, *Lactobacillus delbrueckii* sub sp. *bulgaricus*, *Lactobacillus gasseri* and *Lactobacillus crispatus* as illustrated in Figure 3.

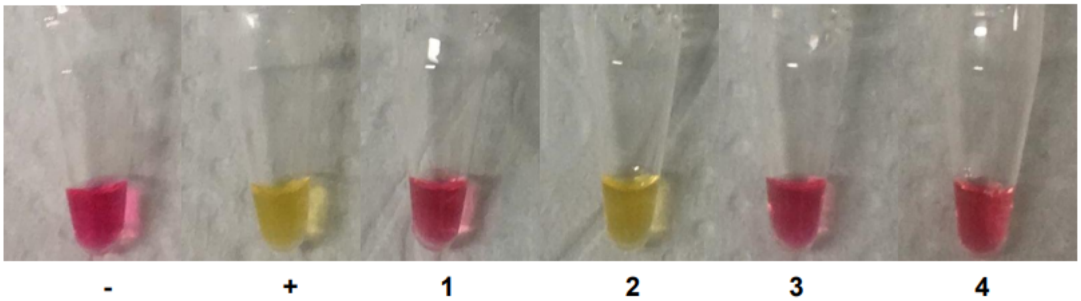

Figure 3: Validation results of the Loop-mediated isothermal amplification method test for the citizen science practical. - negative controle, + positive controle, 1 *L. plantarum*, 2 *L. crispatus*, 3 *L. delbrueckii* sub sp.*bulgaricus*, 4 *L. gasseri*.

11 Furthermore, we tested 134 colonies of the vaginal isolates obtained during the practical and compared the outcome of the LAMP test with a colony PCR using *L. crispatus*-specific primers [4].

- A total of 61 colonies showed a 145 bp band in the colony PCR analysis, matching 100% with the positive outcome of the LAMP test.

Isolation and cultivation

3d

12 For *L. crispatus* isolation we used Tryptic Soy Broth (TSB) agar plates supplemented with 10% horse serum, 1 g/L tween 80 and 10 g/L glucose at pH 5 adjusted it with 10% acetic acid as
previously described [5].

13 To ensure that each participant can successfully isolate *L. crispatus* colonies, despite substantial variation in bacterial load, each citizen scientist inoculated two TSB plates.

13.1 Inoculate the first plate with a large droplet application via a Pasteur Pipet. Spread the sample evenly over the surface using L-spreaders.

13.2 Inoculate the second plate with a small droplet using an inoculating loop for the streak plate method (See Supplemental file 9: Appendix I. Section on Sample Plating from the Lab Journal, for the instructions provided to the donors).

- Figure 4 illustrates the difference in bacterial growth between two citizen scientists on Tryptic Soy Agar.

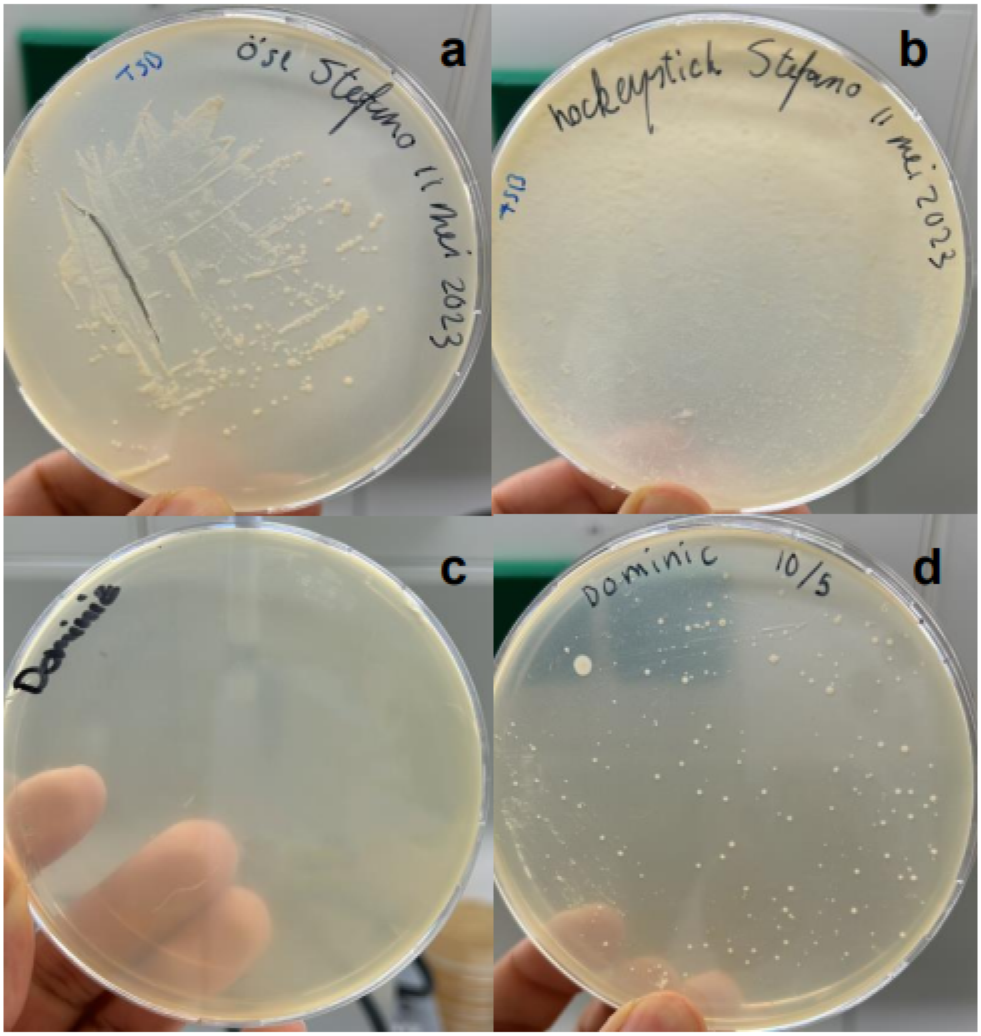

Figure 4: Variation in bacterial growth between vaginal swabs of two citizen scientists on Tryptic Soy Agar.
a) and c) were inoculated using a loop.
b) and d) were inoculated using an L-spreader

14 Incubate the plates anaerobically at 37 °C for 48:00:00 - 72:00:00 using Oxoid™ AnaeroGen(Thermo Fischer Scientific, Waltham, Massachusetts, USA) system. 5d

14.1 Select the colonies based on colony morphology which include being round, creamy white, with a raised central area, and having a slightly rough texture with irregular edges.

- Additionally, we used reference plates showing the morphology of *L. crispatus* along with other known lactobacilli including *L. gasseri*, *L. plantarum* and *L. delbruecki* subsp. *bulgaricus*.

14.2 Later on, we confirmed the identity of the selected colonies with the LAMP test.

14.3 Positive colonies are re-streaked on MRS plates.

- Alongside choosing their own pseudonym, participants are also able to name their *L. crispatus* isolate.

Protocol references

1. Higashide S, Cho O, Matsuda Y, Ogishima D, Kurakado S, Sugita T., Rapid detection of Lactobacillus crispatus and Lactobacillus iners in vaginal specimens by loop-mediated isothermal amplification. Journal of Microbiological Methods. 2019, Vol. 158, pp. 18-20.

2. https://www.neb.com/en/faqs/2020/06/25/what-if-my-colorimetric-lamp-mix-is-orange-instead-of-pink-prior-to-amplification. Accessed on 3 February 2024.

3. Tanner, N.; Zhang, Y.; Evans, T. Visual detection of isothermal nucleic acid amplification using pH-sensitive dyes. BioTechniques. 2018; 58(2):59-68.

4. Kusters JG, Reuland EA, Bouter S, Koenig P, Dorigo-Zetsma JW. A multiplex real-time PCR assay for routine diagnosis of bacterial vaginosis. European Journal of Clinical Microbiology & Infectious Diseases. 2015; 34(9):1779–1785.

5. van der Veer C, Hertzberger RY, Bruisten SM, Tytgat HLP, Swanenburg J, de Kat Angelino-Bart A, Schuren F, Molenaar D, Reid G, de Vries H, Kort R. Comparative genomics of human Lactobacillus crispatus isolates reveals genes for glycosylation and glycogen degradation: implications for in vivo dominance of the vaginal microbiota. Microbiome. 2019 Mar 29;7(1):49.
